# Acute Anterior Thigh Compartment Syndrome in a Young Mixed Martial Arts Fighter

**DOI:** 10.7759/cureus.52820

**Published:** 2024-01-23

**Authors:** Yusuf Bukhamas, Mohamed Elmasri, Abdullah M Alasiri, Abdulla Aljawder

**Affiliations:** 1 Orthopedic Surgery, King Hamad University Hospital, Busaiteen, BHR; 2 Orthopedic Surgery, National Guard Health Affairs Hospital Dammam, Dammam, SAU

**Keywords:** orthopedics and trauma, trauma, youth athlete, mixed martial arts, thigh compartment syndrome

## Abstract

Acute compartment syndrome (ACS) of the thigh is an uncommon injury, and diagnosis of such cases can be easily delayed or even missed due to the rare nature of this condition.

We present a case of ACS of the thigh in a young, healthy mixed martial arts (MMA) semi-professional athlete with no history of previous medical illnesses and normal coagulation.

This MMA fighter sustained a direct blow to the anterolateral aspect of his left thigh with a strong kick during a sparring match with his opponent.

After early surgical fasciotomy, this athlete returned to his pre-injury state and athletic performance within six to eight months postoperatively. Our literature review asserts that young athletic males with high muscle mass, engaging in contact sports, are at a higher risk of developing ACS of the thigh.

## Introduction

Acute compartment syndrome (ACS) of the thigh is one of the main orthopedic emergencies that can lead to debilitating sequelae such as amputation and even death. The most common cause is trauma, leading to damage to underlying muscles and blood vessels within an osseofascial compartment. This damage results in increased intra-compartmental pressure due to edema and swelling, while the fascia enveloping the compartment limits expansion. This, in turn, leads to a significant reduction in blood perfusion to the muscles and nerves, eventually resulting in ischemia and soft tissue necrosis.

ACS is often associated with lower leg compartments and the forearm, whereas ACS of the thigh is quite rare. This is mainly because thigh compartments are large and surrounded by an expansile fascia with the capacity to dilate, readily accommodating expansion. Most common thigh ACS occurs in the anterior compartment due to the susceptibility of this site to injury.

The etiology of such conditions is quite diverse. Some reported cases of ACS of the thigh were attributed to various factors, including traumatic injuries - both with and without fractures - exercise, deep vein thrombosis, drug-induced toxicity (including anticoagulation therapy), venomous snake bites, vascular injuries, and postsurgical complications.

Traumatic ACS of the thigh was previously reported in football, handball, rugby, and direct contact sports involving sparing such as kickboxing, mixed martial arts (MMA), and karate.

ACS of the thigh is a rare condition in sports with scarce epidemiological evidence. Limited agreement is available in the medical field regarding the exact diagnostic criteria and the most suitable treatment strategy or plan for such conditions.

## Case presentation

A young healthy 19-year-old male, MMA athlete with a history of a direct blow to the anterolateral aspect of the left thigh from his opponent with a strong kick during an MMA fight. 

The patient presented with a complaint of moderate pain associated with swelling in the thigh extending to the knee. There was a restricted range of motion in the knee joint, and weight-bearing was painfully difficult, resulting in a noticeable limp.

The patient was admitted for monitoring and observation, additional investigations, and pain control. 

During admission, the pain progressively worsened and was not alleviated by potent analgesia, even disturbing the patient's sleep. 

Examination revealed gross swelling of the left thigh, an immensely tense thigh compartment, tenderness on palpation, a positive passive stretch test indicating increased pain, and palpable distal pulses with no neurovascular compromise. The initial measurement of the left thigh circumference was 2 cm greater than the contralateral thigh, and during admission, a discrepancy of 6 cm compared to the contralateral limb was observed upon establishing the clinical diagnosis of compartment syndrome.

The patient's past medical and surgical history was unremarkable, with no previous hospital admissions. Social and family history was insignificant.

Investigations included an X-ray of the left femur, which showed no fractures. Additionally, a left femur/thigh MRI was obtained, revealing findings suggestive of a high-grade injury with an intramuscular hematoma measuring about 8.5 cm x 3 cm in the vastus lateralis muscle (musculotendinous junction grade 3 according to the British Athletic Muscle Injury Classification).

Treatment

**Figure 1:**
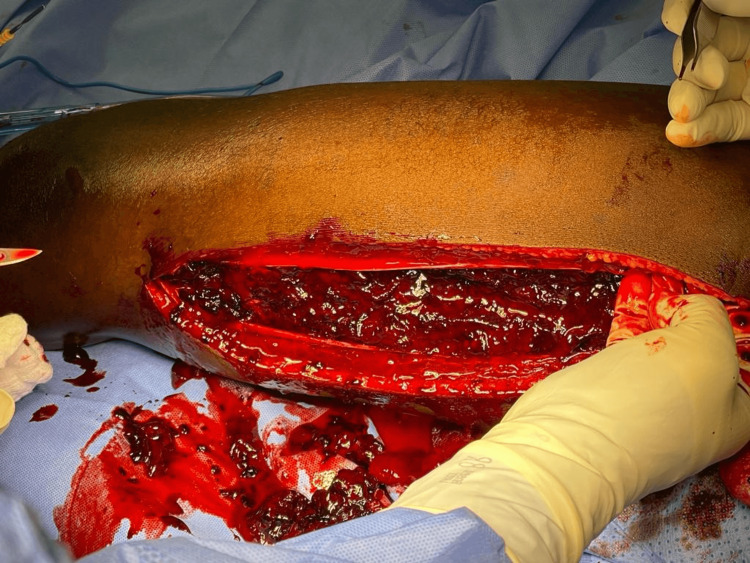
Emergency surgical fasciotomy of the left thigh.

Upon establishing a diagnosis, the patient was urgently taken to the operating theater for emergency fasciotomy and underwent surgical fasciotomy of the left thigh (Figure 1).

**Figure 2:**
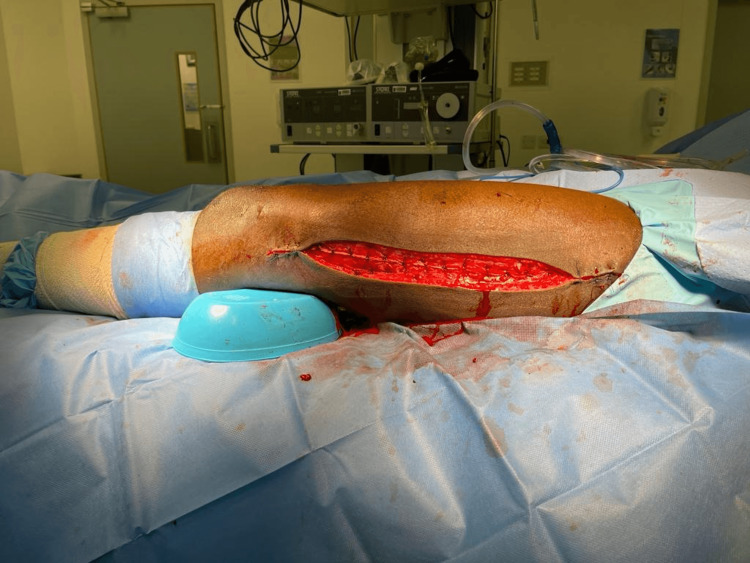
Left thigh wound second look, washout, and partial wound closure.

Two days later, the patient underwent a left thigh wound second look, washout, and partial wound closure (Figure 2).

Three days later, a third look with complete wound closure was performed.

Postoperative rehabilitation and follow-up

Postoperatively, the patient was followed up for wound review, and the patient's level of activity gradually improved with physiotherapy. The patient was able to return to his daily life activities six months postoperatively. 

## Discussion

ACS of the thigh is a rare diagnosis [1], typically linked to motor vehicle accidents [1]. However, it has also been previously reported as a consequence of trauma in sports [2-8].

Early diagnosis and treatment of ACS of the thigh is critical to avoid irreversible tissue damage. This is of profound significance, especially for competitive athletes. Cardinal signs and symptoms of ACS include the 6P's of pallor, paresis, paresthesia, diminished pulse, increased compartment pressure, and pain out of proportion with injury. Measurement of compartment pressures is useful to establish the diagnosis of ACS of the thigh and helps to avoid delayed or missing diagnosis [9].

However, the precise threshold pressures that are diagnostic and indicate intervention remain unclear. Basal intramuscular pressure is 0-8 mmHg [2], while pain and paresthesia occur at 20-30 mmHg [9]. Previous studies suggested that absolute compartment pressures ranging from 30 to 50 mmHg are diagnostic of ACS and indicative of intervention [1]. Nevertheless, most of this evidence was based on intra-compartmental pressures of the lower leg and forearm. The thigh compartments are anatomically distinct from lower leg and forearm compartments, and the applicability of these threshold values to ACS of the thigh remains undetermined [9].

Pain out of proportion with injury is the most common clinical indication used in diagnosing ACS. Even though pain out of proportion has been described as a reliable indicator of ACS of the thigh in some circumstances [9], the pain may be less prominent as the femoral nerve is not completely enclosed within the anterior compartment [5]. Diminished pain can also be due to underlying nerve damage [9]. Thigh contusions are very common in contact sports, and differentiating between a mild contusion and ACS of the thigh can be difficult. Unlike other parts of the body, ACS of the thigh is rarely associated with fracture [9] and has not been reported in the sporting context [2-6,8]. Without an obvious history of trauma and severe pain, a diagnosis of ACS of the thigh can be easily missed [9].

The rareness of ACS of the thigh makes it tough to study, and the most suitable treatment for this condition is yet unclear. A prompt fasciotomy with the concurrent evacuation of hematoma is frequently advocated to restore normal capillary perfusion [5]. Poor outcomes have been associated with delayed surgical intervention [9]. Of the sports-related cases reported, 10 of 16 cases underwent fasciotomy after presenting with absolute compartment pressures ranging from 39 to 120 mmHg [3-6,8]. No surgical complications were reported, and 4 out of 10 cases returned to their respective sports. Significantly, 5 out of 16 athletes in the reported cases resumed playing after sustaining the injury [1,5], potentially exacerbating the condition and narrowing the window for early intervention.

It was argued in some studies that surgical intervention sometimes results in unnecessary harm. Unlike fasciotomies, nonsurgical treatment does not lead to possible complications like decreased range of motion due to adhesions [2,8]. Also, postoperative infections are common. That said, the risks of delaying surgery are far greater than the risks associated with the procedure.

The patient in the case we are presenting currently competes at a high level, thanks to uncomplicated surgical intervention alongside intensive physiotherapy [9].

It is important to discuss a few risk factors that could alert clinicians and help them consider this uncommon diagnosis as a differential diagnosis. In a sporting context, 100% of the reported cases were males, and their mean age was 22.8 years [2-6,8]. Another thing to note, larger muscles seem to increase the chances of ACS in the thigh [1]. When muscles grow, compartment sizes remain the same. This, in turn, decreases the space available for swelling after a trauma, which increases the risk of ACS. In conclusion, we should watch out for ACS of the thigh in young athletic males with substantial muscle mass who are involved in contact sports.

The rare nature of this diagnosis leaves plenty of room for doubt regarding the ideal ways to diagnose and treat ACS of the thigh. Despite this, it is crucial to exercise utmost care in diagnosing and managing it early to prevent severe consequences, such as premature career endings or even death [9].

## Conclusions

ACS of the thigh is an uncommon condition, and diagnosis may be delayed or missed mainly in the sporting context due to the insidious progression of this rare condition.

A very high index of suspicion must be present for early diagnosis, especially in cases of traumatic thigh contusions in the absence of fractures, particularly in young athletes with high muscle mass.

Early diagnosis, following urgent surgical fasciotomy, and proper rehabilitation are key factors for patients to recover and achieve a pre-trauma level of performance without severe complications.
